# Supplementary material for: Dysregulated activities of proline-specific enzymes in septic shock patients (sepsis-2)
Source: PLoS One. 2020 Apr 21;15(4):e0231555. doi: 10.1371/journal.pone.0231555 (PMC7173796; doi:10.1371/journal.pone.0231555)
Supplement: S1 Fig — The plasma activity of DPP4 (A), FAP (B), PRCP (C) and PREP (D), expressed in U/L (median with interquartile range), measured in a healthy control group (n = 29–30) and the ICU control group (n = 22). Differences between the two control groups were tested using Mann-Whitney U tests. Abbreviations used: DPP4: dipeptidyl peptidase 4; FAP: fibroblast activation protein α; NS: non-significant; PRCP: prolylcarboxypeptidase; PREP: prolyl oligopeptidase; U/L: units per liter. (DOCX) [file pone.0231555.s001.docx]

## S1 Fig: Plasma activity of DPP4, FAP, PRCP and PREP in healthy and ICU controls.


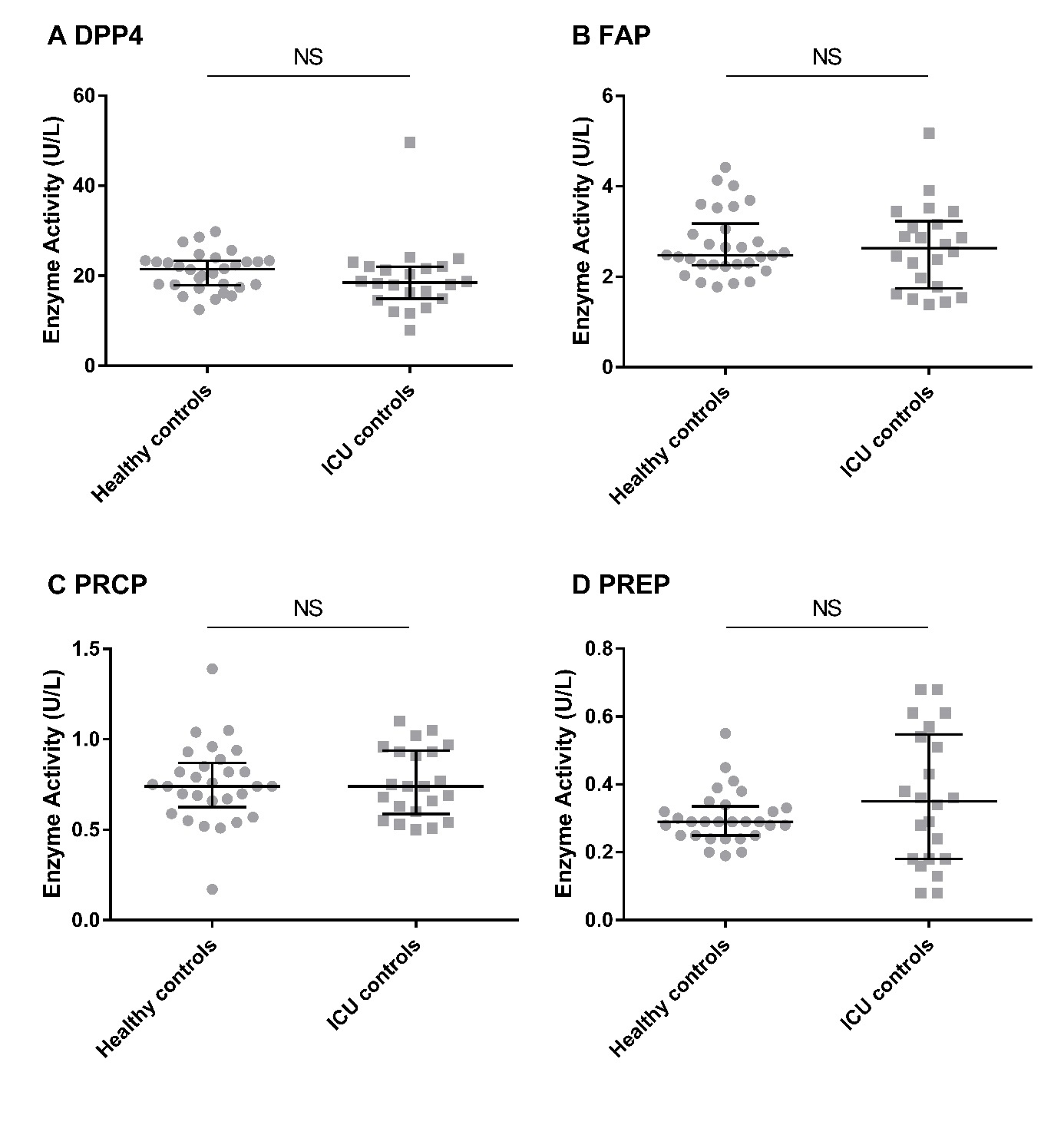


The plasma activity of DPP4 (A), FAP (B), PRCP (C) and PREP (D), expressed in U/L (median with interquartile range), measured in a healthy control group (n = 29-30) and the ICU control group (n = 22). Differences between the two control groups were tested using Mann-Whitney U tests. Abbreviations used: DPP4: dipeptidyl peptidase 4; FAP: fibroblast activation protein α; NS: non-significant; PRCP: prolylcarboxypeptidase; PREP: prolyl oligopeptidase; U/L: units per liter.
