# Supplementary material for: Dysregulated activities of proline-specific enzymes in septic shock patients (sepsis-2)
Source: PLoS One. 2020 Apr 21;15(4):e0231555. doi: 10.1371/journal.pone.0231555 (PMC7173796; doi:10.1371/journal.pone.0231555)
Supplement: S2 Fig — Plot of the first two principal components scores for the ICU control group and the healthy control group, based upon PREP, PRCP, FAP and DPP4. Abbreviations used: ICU; intensive care unit; PC: principal component. (DOCX) [file pone.0231555.s002.docx]

## S2 Fig: Principal Component Analysis


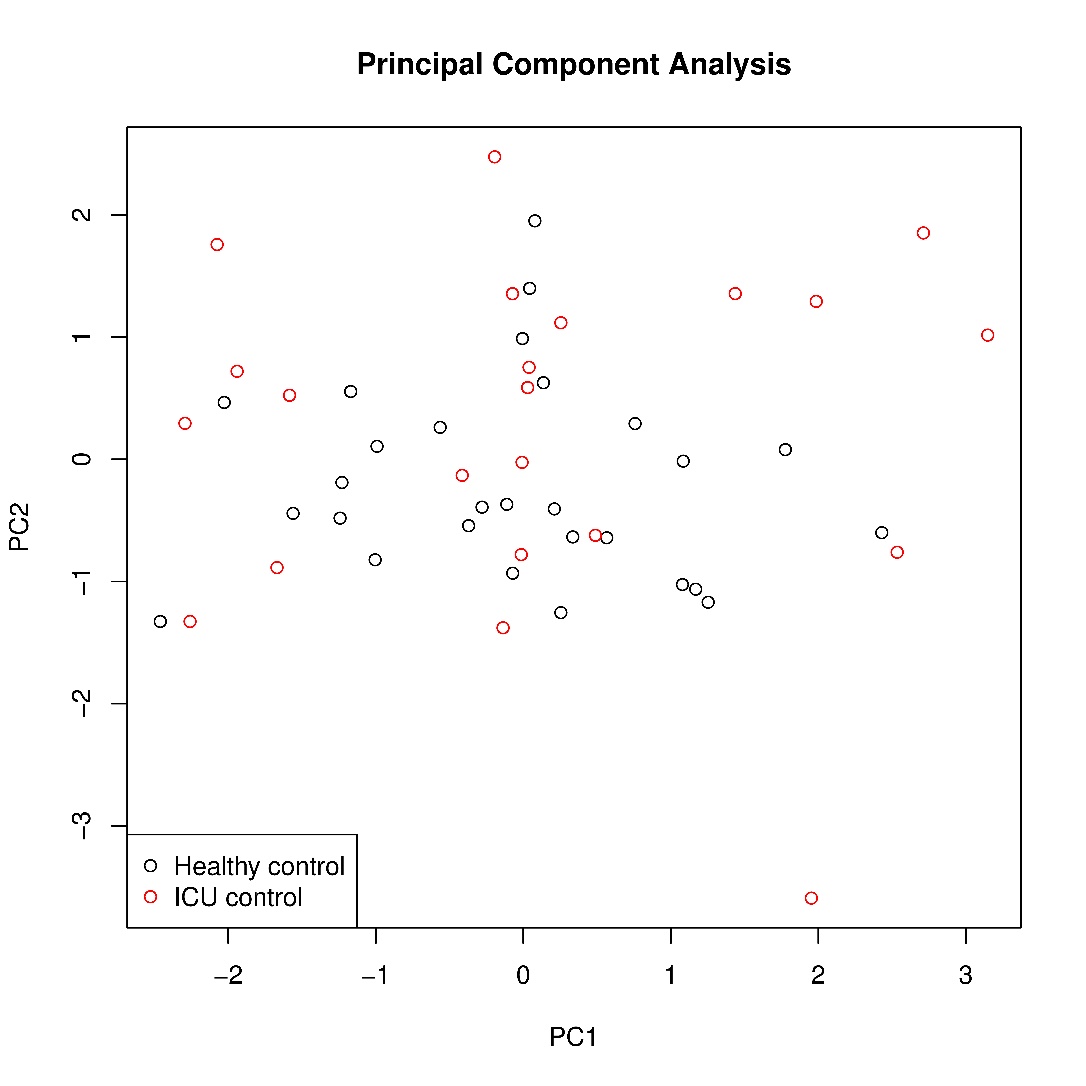


Plot of the first two principal components scores for the ICU control group and the healthy control group, based upon PREP, PRCP, FAP and DPP4. Abbreviations used: ICU; intensive care unit; PC: principal component.
