# Supplementary material for: Dysregulated activities of proline-specific enzymes in septic shock patients (sepsis-2)
Source: PLoS One. 2020 Apr 21;15(4):e0231555. doi: 10.1371/journal.pone.0231555 (PMC7173796; doi:10.1371/journal.pone.0231555)
Supplement: S3 Fig — (DOCX) [file pone.0231555.s003.docx]

**S3 Fig: Receiver operating characteristic (ROC) curves of the combination FAP and DPP4 and FAP with PREP.**

| **A FAP + DPP4**  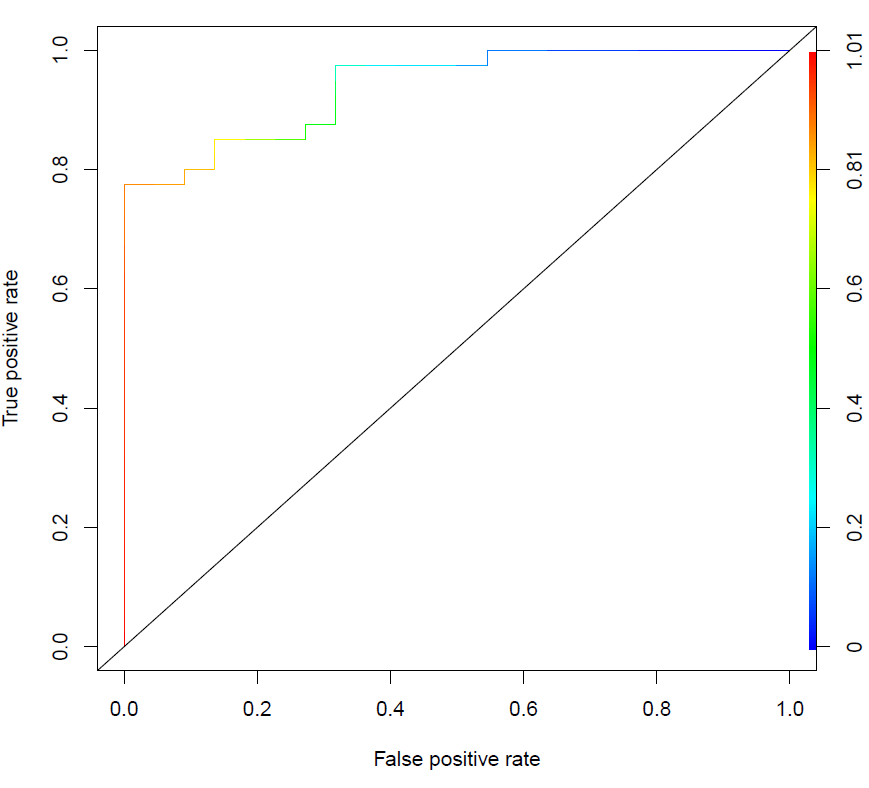  **AUC = 0.94** |
| --- |
| **B FAP + PREP**  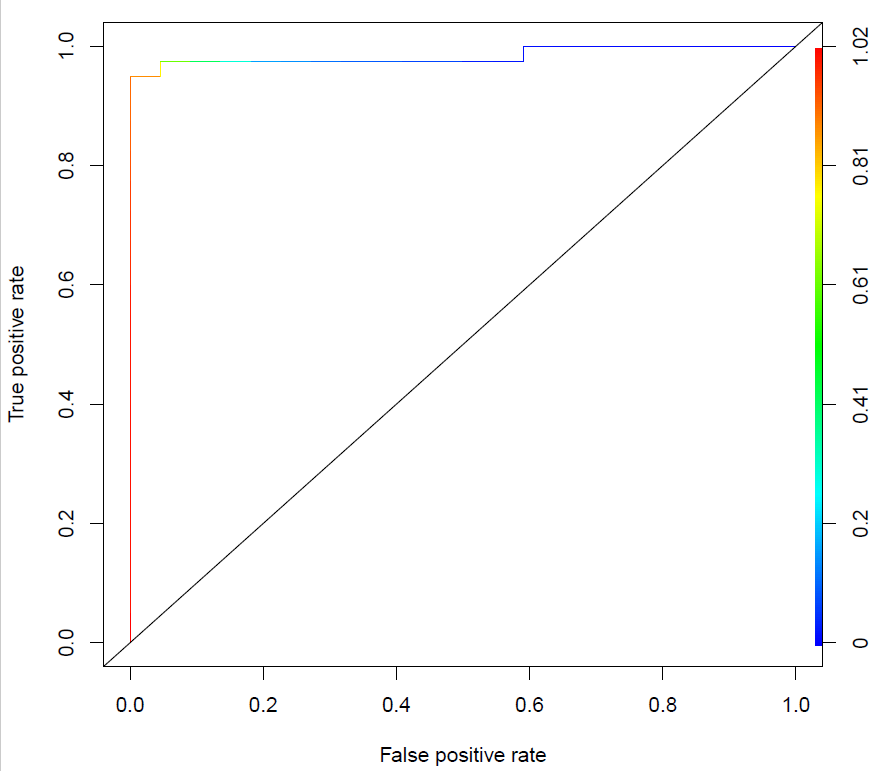  **AUC = 1** |
