# Supplementary material for: Dysregulated activities of proline-specific enzymes in septic shock patients (sepsis-2)
Source: PLoS One. 2020 Apr 21;15(4):e0231555. doi: 10.1371/journal.pone.0231555 (PMC7173796; doi:10.1371/journal.pone.0231555)

## S4 Fig: Visual representation of the statistical analysis of the studied associations.

(A) Dot plot of the log-transformed *p*-value for the interaction (*p*Int) for the longitudinally measured parameters. The dashed line indicates a *p*-value of 0.01. Significant interactions were obtained for PRCP with total bilirubin and TNFα. P-values are based upon linear mixed models. (B) Dot plot of the log-transformed *p*-value for the main effect (*p*Main) for the longitudinally measured parameters. In case of a significant interaction between time and the parameter, no main effect *p*-value is shown in the dot plot. The dashed line indicates a *p*-value of 0.01. Linear mixed models were fitted to obtain the *p*-values. (C) R² for the longitudinally measured parameters. R² is only depicted when *p*Int ˃ 0.01. P-values are based upon linear mixed models. ○ DPP4; + PRCP; △ FAP; ● PREP. Abbreviations used: DPP4: dipeptidyl peptidase 4; FAP: fibroblast activation protein α; I-FABP: intestinal fatty acid-binding protein; IFNγ: interferon γ; IL: interleukin; IL-1RA: interleukin-1 receptor antagonist; MAP: mean arterial pressure; NIR, noradrenalin infusion rate; PaO_2_/FiO_2_ ratio: ratio of arterial oxygen partial pressure to fractional inspired oxygen; PRCP: prolylcarboxypeptidase; PREP: prolyl oligopeptidase; TNFα: tumor necrosis factor α.


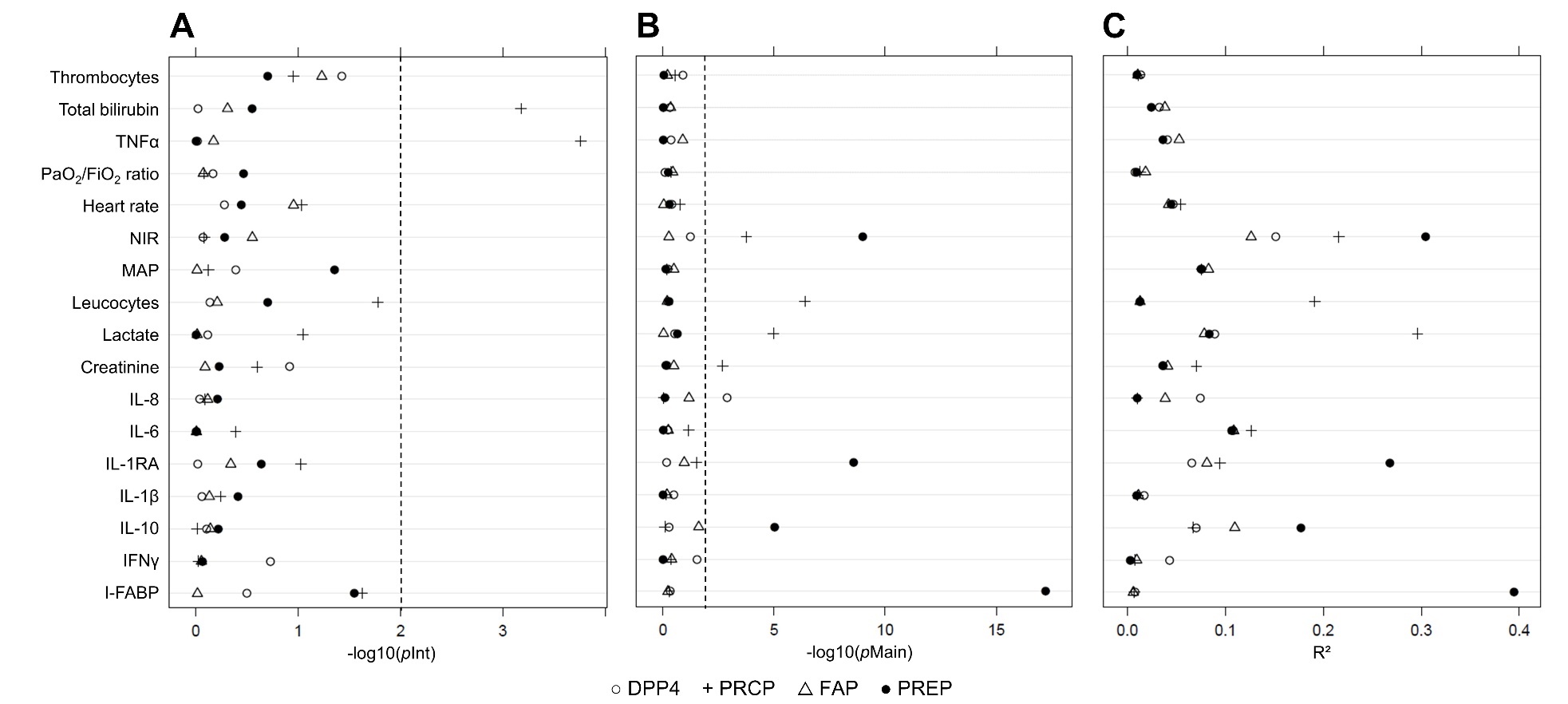

Supplement: S4 Fig — (A) Dot plot of the log-transformed p-value for the interaction (pInt) for the longitudinally measured parameters. The dashed line indicates a p-value of 0.01. Significant interactions were obtained for PRCP with total bilirubin and TNFα. P-values are based upon linear mixed models. (B) Dot plot of the log-transformed p-value for the main effect (pMain) for the longitudinally measured parameters. In case of a significant interaction between time and the parameter, no main effect p-value is shown in the dot plot. The dashed line indicates a p-value of 0.01. Linear mixed models were fitted to obtain the p-values. (C) R² for the longitudinally measured parameters. R2 is only depicted when pInt ˃ 0.01. P-values are based upon linear mixed models. ○ DPP4; + PRCP; △ FAP; ● PREP. Abbreviations used: DPP4: dipeptidyl peptidase 4; FAP: fibroblast activation protein α; I-FABP: intestinal fatty acid-binding protein; IFNγ: interferon γ; IL: interleukin; IL-1RA: interleukin-1 receptor antagonist; MAP: mean arterial pressure; NIR, noradrenalin infusion rate; PaO2/FiO2 ratio: ratio of arterial oxygen partial pressure to fractional inspired oxygen; PRCP: prolylcarboxypeptidase; PREP: prolyl oligopeptidase; TNFα: tumor necrosis factor α. (DOCX) [file pone.0231555.s004.docx]
