# Supplementary material for: Dysregulated activities of proline-specific enzymes in septic shock patients (sepsis-2)
Source: PLoS One. 2020 Apr 21;15(4):e0231555. doi: 10.1371/journal.pone.0231555 (PMC7173796; doi:10.1371/journal.pone.0231555)
Supplement: S1 Table — A total of 22 patients were included for this study. (DOCX) [file pone.0231555.s005.docx]

## S1 Table: Characteristics of the non-septic shock ICU control group.

A total of 22 patients were included for this study.

| **Age** | **Gender** | **Surgery** | **Remarks** |
| --- | --- | --- | --- |
| 62 | Female | Clipping aneurysma cerebri |  |
| 41 | Male | Tumor Cerebri | Dexamethasone given during surgery. |
| 58 | Female | Vestibular schwannoma |  |
| 49 | Female | Tumor Cerebri |  |
| 66 | Female | Tumor Cerebri |  |
| 60 | Female | Vestibular schwannoma |  |
| 43 | Male | Vestibular schwannoma |  |
| 38 | Male | Tumor Cerebri |  |
| 37 | Female | Tumor Cerebri | Dexamethasone given during surgery. |
| 60 | Female | Vestibular schwannoma |  |
| 65 | Female | Clipping aneurysma cerebri |  |
| 64 | Male | Tumor Cerebri |  |
| 60 | Male | Vestibular schwannoma |  |
| 50 | Female | Clipping aneurysma cerebri |  |
| 32 | Female | Tumor Cerebri |  |
| 51 | Male | Tumor Cerebri |  |
| 50 | Male | Tumor Cerebri | Dexamethasone given during surgery. |
| 53 | Male | Tumor Cerebri |  |
| 64 | Female | Tumor Cerebri |  |
| 31 | Male | Clipping aneurysma cerebri |  |
| 33 | Female | Tumor Cerebri |  |
| 46 | Female | Tumor Cerebri |  |
