# Supplementary material for: Dysregulated activities of proline-specific enzymes in septic shock patients (sepsis-2)
Source: PLoS One. 2020 Apr 21;15(4):e0231555. doi: 10.1371/journal.pone.0231555 (PMC7173796; doi:10.1371/journal.pone.0231555)
Supplement: S2 Table — Enzymatic activities were determined in EDTA-plasma of septic shock patients on day 1, 3, 5 and 7 and non-septic shock ICU control patients. Data are represented as median (interquartile range). Abbreviations used: DPP4: dipeptidyl peptidase 4; FAP: fibroblast activation protein α; PRCP: prolylcarboxypeptidase; PREP: prolyl oligopeptidase; U/L: units per liter. (DOCX) [file pone.0231555.s006.docx]

## S2 Table: The enzyme activity of DPP4, FAP, PRCP and PREP.

Enzymatic activities were determined in EDTA-plasma of septic shock patients on day 1, 3, 5 and 7 and non-septic shock ICU control patients. Data are represented as median (interquartile range). Abbreviations used: DPP4: dipeptidyl peptidase 4; FAP: fibroblast activation protein α; PRCP: prolylcarboxypeptidase; PREP: prolyl oligopeptidase; U/L: units per liter.

| **Enzyme activity, U/L** | **ICU controls (*n* = 22)** | **Day 1 (*n* = 40)** | **Day 3 (*n* = 40)** | **Day 5 (*n* = 39)** | **Day 7 (*n* = 39)** |
| --- | --- | --- | --- | --- | --- |
| **DPP4** | 18.51 (14.85-22.04) | 9.92 (7.36-15.36) | 9.16 (6.85-11.78) | 9.90 (5.72-12.00) | 10.00 (6.46-11.89) |
| **FAP** | 2.64 (1.74-3.23) | 0.93 (0.68-1.27) | 0.76 (0.57-1.21) | 0.97 (0.65-1.27) | 0.95 (0.62-1.26) |
| **PRCP** | 0.74 (0.59-0.94) | 0.93 (0.71-1.59) | 0.85 (0.61-1.32) | 0.90 (0.62-1.20) | 0.92 (0.74-1.18) |
| **PREP** | 0.35 (0.18-0.55) | 0.85 (0.54-1.32) | 0.63 (0.52-1.06) | 0.87 (0.52-1.11) | 0.74 (0.52-1.01) |
