# Supplementary material for: Dysregulated activities of proline-specific enzymes in septic shock patients (sepsis-2)
Source: PLoS One. 2020 Apr 21;15(4):e0231555. doi: 10.1371/journal.pone.0231555 (PMC7173796; doi:10.1371/journal.pone.0231555)
Supplement: S3 Table — The 1-specificity, sensitivity, negative predictive value (NPV), positive predictive value (PPV), positive likelihood ratio (LR+), negative likelihood ratio (LR-) and Youden index for every cutoff value expressed in U/L for DPP4. For the septic shock patients day 1 was used. Blue indicates the maximum for the Youden index. Red indicates where the sensitivity reaches 1. In the case of DPP4, a value lower than the indicated cutoff would suggest a diagnosis with septic shock. ICU controls: n = 22; Septic shock patients: n = 40. (DOCX) [file pone.0231555.s007.docx]

## S3 Table: Cutoff values receiver operating characteristic curve of dipeptidyl peptidase 4 (DPP4).

The 1-specificity, sensitivity, negative predictive value (NPV), positive predictive value (PPV), positive likelihood ratio (LR+), negative likelihood ratio (LR-) and Youden index for every cutoff value expressed in U/L for DPP4. For the septic shock patients day 1 was used. Blue indicates the maximum for the Youden index. Red indicates where the sensitivity reaches 1. In the case of DPP4, a value lower than the indicated cutoff would suggest a diagnosis with septic shock.
ICU controls: n = 22; Septic shock patients: n = 40.

| **Cutoff value, U/L** | **1-specificity** | **Sensitivity** | **NPV** | **PPV** | **LR+** | **LR-** | **Youden index** |
| --- | --- | --- | --- | --- | --- | --- | --- |
| 49.63 | 1.00 | 1.00 | NA | 0.65 | 1.00 | NA | 0.00 |
| 24.14 | 0.95 | 1.00 | 1.00 | 0.66 | 1.05 | 0.00 | 0.05 |
| 23.82 | 0.91 | 1.00 | 1.00 | 0.67 | 1.10 | 0.00 | 0.09 |
| 23.05 | 0.86 | 1.00 | 1.00 | 0.68 | 1.16 | 0.00 | 0.14 |
| 22.06 | 0.82 | 1.00 | 1.00 | 0.69 | 1.22 | 0.00 | 0.18 |
| 22.03 | 0.77 | 0.98 | 0.83 | 0.70 | 1.26 | 0.11 | 0.20 |
| 21.60 | 0.73 | 0.98 | 0.86 | 0.71 | 1.34 | 0.09 | 0.25 |
| 21.30 | 0.68 | 0.98 | 0.88 | 0.72 | 1.43 | 0.08 | 0.29 |
| 20.37 | 0.64 | 0.98 | 0.89 | 0.74 | 1.53 | 0.07 | 0.34 |
| 18.76 | 0.59 | 0.98 | 0.90 | 0.75 | 1.65 | 0.06 | 0.38 |
| 18.70 | 0.55 | 0.98 | 0.91 | 0.76 | 1.79 | 0.06 | 0.43 |
| 18.61 | 0.50 | 0.98 | 0.92 | 0.78 | 1.95 | 0.05 | 0.48 |
| 18.32 | 0.50 | 0.90 | 0.73 | 0.77 | 1.80 | 0.20 | 0.40 |
| 18.06 | 0.45 | 0.90 | 0.75 | 0.78 | 1.98 | 0.18 | 0.45 |
| 17.93 | 0.41 | 0.90 | 0.76 | 0.80 | 2.20 | 0.17 | 0.49 |
| 17.79 | 0.36 | 0.90 | 0.78 | 0.82 | 2.48 | 0.16 | 0.54 |
| 17.31 | 0.36 | 0.88 | 0.74 | 0.81 | 2.41 | 0.20 | 0.51 |
| 16.69 | 0.36 | 0.85 | 0.70 | 0.81 | 2.34 | 0.24 | 0.49 |
| 16.63 | 0.36 | 0.83 | 0.67 | 0.80 | 2.27 | 0.28 | 0.46 |
| 16.52 | 0.36 | 0.80 | 0.64 | 0.80 | 2.20 | 0.31 | 0.44 |
| 16.42 | 0.32 | 0.80 | 0.65 | 0.82 | 2.51 | 0.29 | 0.48 |
| 16.23 | 0.32 | 0.78 | 0.63 | 0.82 | 2.44 | 0.33 | 0.46 |
| 15.58 | 0.27 | 0.78 | 0.64 | 0.84 | 2.84 | 0.31 | 0.50 |
| 14.95 | 0.27 | 0.75 | 0.62 | 0.83 | 2.75 | 0.34 | 0.48 |
| 14.69 | 0.23 | 0.75 | 0.63 | 0.86 | 3.30 | 0.32 | 0.52 |
| 14.56 | 0.23 | 0.73 | 0.61 | 0.85 | 3.19 | 0.36 | 0.50 |
| 13.59 | 0.18 | 0.73 | 0.62 | 0.88 | 3.99 | 0.34 | 0.54 |
| 12.85 | 0.18 | 0.70 | 0.60 | 0.88 | 3.85 | 0.37 | 0.52 |
| 11.96 | 0.14 | 0.70 | 0.61 | 0.90 | 5.13 | 0.35 | 0.56 |
| 11.93 | 0.09 | 0.70 | 0.63 | 0.93 | 7.70 | 0.33 | 0.61 |
| 11.68 | 0.09 | 0.68 | 0.61 | 0.93 | 7.43 | 0.36 | 0.58 |
| 11.41 | 0.05 | 0.68 | 0.62 | 0.96 | 14.85 | 0.34 | 0.63 |
| 11.08 | 0.05 | 0.65 | 0.60 | 0.96 | 14.30 | 0.37 | 0.60 |
| 10.94 | 0.05 | 0.63 | 0.58 | 0.96 | 13.75 | 0.39 | 0.58 |
| 10.50 | 0.05 | 0.60 | 0.57 | 0.96 | 13.20 | 0.42 | 0.55 |
| 10.35 | 0.05 | 0.58 | 0.55 | 0.96 | 12.65 | 0.45 | 0.53 |
| 10.23 | 0.05 | 0.55 | 0.54 | 0.96 | 12.10 | 0.47 | 0.50 |
| 10.18 | 0.05 | 0.53 | 0.53 | 0.95 | 11.55 | 0.50 | 0.48 |
| 9.66 | 0.05 | 0.50 | 0.51 | 0.95 | 11.00 | 0.52 | 0.45 |
| 9.56 | 0.05 | 0.48 | 0.50 | 0.95 | 10.45 | 0.55 | 0.43 |
| 9.47 | 0.05 | 0.45 | 0.49 | 0.95 | 9.90 | 0.58 | 0.40 |
| 9.23 | 0.05 | 0.43 | 0.48 | 0.94 | 9.35 | 0.60 | 0.38 |
| 9.09 | 0.05 | 0.40 | 0.47 | 0.94 | 8.80 | 0.63 | 0.35 |
| 9.02 | 0.05 | 0.38 | 0.46 | 0.94 | 8.25 | 0.65 | 0.33 |
| 8.78 | 0.05 | 0.35 | 0.45 | 0.93 | 7.70 | 0.68 | 0.30 |
| 7.95 | 0.05 | 0.33 | 0.44 | 0.93 | 7.15 | 0.71 | 0.28 |
| 7.87 | 0.00 | 0.33 | 0.45 | 1.00 | ∞ | 0.68 | 0.33 |
| 7.64 | 0.00 | 0.30 | 0.44 | 1.00 | ∞ | 0.70 | 0.30 |
| 7.43 | 0.00 | 0.28 | 0.43 | 1.00 | ∞ | 0.73 | 0.28 |
| 7.34 | 0.00 | 0.25 | 0.42 | 1.00 | ∞ | 0.75 | 0.25 |
| 7.29 | 0.00 | 0.23 | 0.42 | 1.00 | ∞ | 0.78 | 0.23 |
| 6.97 | 0.00 | 0.20 | 0.41 | 1.00 | ∞ | 0.80 | 0.20 |
| 6.93 | 0.00 | 0.18 | 0.40 | 1.00 | ∞ | 0.83 | 0.18 |
| 6.47 | 0.00 | 0.15 | 0.39 | 1.00 | ∞ | 0.85 | 0.15 |
| 6.38 | 0.00 | 0.13 | 0.39 | 1.00 | ∞ | 0.88 | 0.13 |
| 5.98 | 0.00 | 0.10 | 0.38 | 1.00 | ∞ | 0.90 | 0.10 |
| 5.84 | 0.00 | 0.08 | 0.37 | 1.00 | ∞ | 0.93 | 0.08 |
| 4.81 | 0.00 | 0.05 | 0.37 | 1.00 | ∞ | 0.95 | 0.05 |
| 4.67 | 0.00 | 0.03 | 0.36 | 1.00 | ∞ | 0.98 | 0.02 |
| Inf | 0.00 | 0.00 | 0.35 | NA | ∞ | 1.00 | 0.00 |

Abbreviations used: LR+: positive likelihood ratio; LR-: negative likelihood ratio; NA: not applicable; NPV: negative predictive value; PPV: positive predictive value; U/L, units per liter.
