# Supplementary material for: Dysregulated activities of proline-specific enzymes in septic shock patients (sepsis-2)
Source: PLoS One. 2020 Apr 21;15(4):e0231555. doi: 10.1371/journal.pone.0231555 (PMC7173796; doi:10.1371/journal.pone.0231555)
Supplement: S5 Table — The 1-specificity, sensitivity, negative predictive value (NPV), positive predictive value (PPV), positive likelihood ratio (LR+), negative likelihood ratio (LR-) and Youden index for every cutoff value expressed in U/L for PRCP. For the septic shock patients day 1 was used. Blue indicates the maximum for the Youden index. Two maxima were obtained for PRCP, in this case the cutoff that would yield the highest sensitivity was selected, as that would identify more patients with septic shock. Red indicates where the sensitivity reaches 1. In the case of PRCP, a value higher than the indicated cutoff would suggest a diagnosis with septic shock. ICU controls: n = 22; Septic shock patients: n = 40. (DOCX) [file pone.0231555.s009.docx]

## S5 Table: Cutoff values receiver operating characteristic curve of prolylcarboxypeptidase (PRCP).

The 1-specificity, sensitivity, negative predictive value (NPV), positive predictive value (PPV), positive likelihood ratio (LR+), negative likelihood ratio (LR-) and Youden index for every cutoff value expressed in U/L for PRCP. For the septic shock patients day 1 was used. Blue indicates the maximum for the Youden index. Two maxima were obtained for PRCP, in this case the cutoff that would yield the highest sensitivity was selected, as that would identify more patients with septic shock. Red indicates where the sensitivity reaches 1. In the case of PRCP, a value higher than the indicated cutoff would suggest a diagnosis with septic shock. ICU controls: n = 22; Septic shock patients: n = 40.

| **Cutoff value, U/L** | **1-specificity** | **Sensitivity** | **NPV** | **PPV** | **LR+** | **LR-** | **Youden index** |
| --- | --- | --- | --- | --- | --- | --- | --- |
| 0.17 | 1.00 | 1.00 | NA | 0.65 | 1.00 | NA | 0.00 |
| 0.43 | 1.00 | 0.98 | 0.00 | 0.64 | 0.98 | NA | -0.03 |
| 0.50 | 1.00 | 0.95 | 0.00 | 0.63 | 0.95 | NA | -0.05 |
| 0.51 | 0.95 | 0.95 | 0.33 | 0.64 | 1.00 | 1.10 | 0.00 |
| 0.53 | 0.91 | 0.95 | 0.50 | 0.66 | 1.05 | 0.55 | 0.04 |
| 0.54 | 0.86 | 0.95 | 0.60 | 0.67 | 1.10 | 0.37 | 0.09 |
| 0.55 | 0.82 | 0.95 | 0.67 | 0.68 | 1.16 | 0.28 | 0.13 |
| 0.57 | 0.77 | 0.95 | 0.71 | 0.69 | 1.23 | 0.22 | 0.18 |
| 0.59 | 0.77 | 0.93 | 0.63 | 0.69 | 1.20 | 0.33 | 0.15 |
| 0.60 | 0.77 | 0.90 | 0.56 | 0.68 | 1.16 | 0.44 | 0.13 |
| 0.63 | 0.73 | 0.90 | 0.60 | 0.69 | 1.24 | 0.37 | 0.17 |
| 0.66 | 0.68 | 0.90 | 0.64 | 0.71 | 1.32 | 0.31 | 0.22 |
| 0.67 | 0.64 | 0.88 | 0.62 | 0.71 | 1.38 | 0.34 | 0.24 |
| 0.68 | 0.64 | 0.85 | 0.57 | 0.71 | 1.34 | 0.41 | 0.21 |
| 0.69 | 0.59 | 0.83 | 0.56 | 0.72 | 1.40 | 0.43 | 0.23 |
| 0.70 | 0.55 | 0.78 | 0.53 | 0.72 | 1.42 | 0.49 | 0.23 |
| 0.72 | 0.55 | 0.75 | 0.50 | 0.71 | 1.38 | 0.55 | 0.20 |
| 0.73 | 0.55 | 0.73 | 0.48 | 0.71 | 1.33 | 0.60 | 0.18 |
| 0.74 | 0.55 | 0.70 | 0.45 | 0.70 | 1.28 | 0.66 | 0.15 |
| 0.75 | 0.45 | 0.68 | 0.48 | 0.73 | 1.49 | 0.60 | 0.22 |
| 0.77 | 0.41 | 0.68 | 0.50 | 0.75 | 1.65 | 0.55 | 0.27 |
| 0.78 | 0.36 | 0.68 | 0.52 | 0.77 | 1.86 | 0.51 | 0.31 |
| 0.81 | 0.36 | 0.65 | 0.50 | 0.76 | 1.79 | 0.55 | 0.29 |
| 0.85 | 0.36 | 0.60 | 0.47 | 0.75 | 1.65 | 0.63 | 0.24 |
| 0.87 | 0.36 | 0.58 | 0.45 | 0.74 | 1.58 | 0.67 | 0.21 |
| 0.88 | 0.36 | 0.55 | 0.44 | 0.73 | 1.51 | 0.71 | 0.19 |
| 0.89 | 0.36 | 0.53 | 0.42 | 0.72 | 1.44 | 0.75 | 0.16 |
| 0.91 | 0.36 | 0.50 | 0.41 | 0.71 | 1.38 | 0.79 | 0.14 |
| 0.93 | 0.32 | 0.50 | 0.43 | 0.74 | 1.57 | 0.73 | 0.18 |
| 0.96 | 0.23 | 0.50 | 0.46 | 0.80 | 2.20 | 0.65 | 0.27 |
| 0.97 | 0.18 | 0.48 | 0.46 | 0.83 | 2.61 | 0.64 | 0.29 |
| 1.02 | 0.14 | 0.48 | 0.48 | 0.86 | 3.48 | 0.61 | 0.34 |
| 1.03 | 0.09 | 0.48 | 0.49 | 0.90 | 5.23 | 0.58 | 0.38 |
| 1.05 | 0.09 | 0.43 | 0.47 | 0.89 | 4.68 | 0.63 | 0.33 |
| 1.07 | 0.05 | 0.43 | 0.48 | 0.94 | 9.35 | 0.60 | 0.38 |
| 1.09 | 0.05 | 0.40 | 0.47 | 0.94 | 8.80 | 0.63 | 0.35 |
| 1.10 | 0.05 | 0.38 | 0.46 | 0.94 | 8.25 | 0.65 | 0.33 |
| 1.19 | 0.00 | 0.35 | 0.46 | 1.00 | ∞ | 0.65 | 0.35 |
| 1.22 | 0.00 | 0.33 | 0.45 | 1.00 | ∞ | 0.68 | 0.33 |
| 1.30 | 0.00 | 0.30 | 0.44 | 1.00 | ∞ | 0.70 | 0.30 |
| 1.45 | 0.00 | 0.28 | 0.43 | 1.00 | ∞ | 0.73 | 0.28 |
| 1.63 | 0.00 | 0.25 | 0.42 | 1.00 | ∞ | 0.75 | 0.25 |
| 1.74 | 0.00 | 0.23 | 0.42 | 1.00 | ∞ | 0.78 | 0.23 |
| 1.88 | 0.00 | 0.20 | 0.41 | 1.00 | ∞ | 0.80 | 0.20 |
| 1.89 | 0.00 | 0.18 | 0.40 | 1.00 | ∞ | 0.83 | 0.18 |
| 2.56 | 0.00 | 0.15 | 0.39 | 1.00 | ∞ | 0.85 | 0.15 |
| 2.81 | 0.00 | 0.13 | 0.39 | 1.00 | ∞ | 0.88 | 0.13 |
| 2.93 | 0.00 | 0.10 | 0.38 | 1.00 | ∞ | 0.90 | 0.10 |
| 3.06 | 0.00 | 0.08 | 0.37 | 1.00 | ∞ | 0.93 | 0.08 |
| 3.63 | 0.00 | 0.05 | 0.37 | 1.00 | ∞ | 0.95 | 0.05 |
| 4.31 | 0.00 | 0.03 | 0.36 | 1.00 | ∞ | 0.98 | 0.02 |
| Inf | 0.00 | 0.00 | 0.35 | NA | ∞ | 1.00 | 0.00 |

Abbreviations used: LR+: positive likelihood ratio; LR-: negative likelihood ratio; NA: not applicable; NPV: negative predictive value; PPV: positive predictive value; U/L, units per liter.
