# Supplementary material for: Dysregulated activities of proline-specific enzymes in septic shock patients (sepsis-2)
Source: PLoS One. 2020 Apr 21;15(4):e0231555. doi: 10.1371/journal.pone.0231555 (PMC7173796; doi:10.1371/journal.pone.0231555)
Supplement: S1 Appendix — (DOCX) [file pone.0231555.s011.docx]

**S1 Appendix: Dysregulated activities of proline-specific enzymes in septic shock patients (sepsis-2)**

# Materials and Methods

## Healthy control samples and blood sampling

30 healthy individuals were additionally included after written informed consent. These controls were recruited among employees of the University of Antwerp and their families and friends. The study was approved by the Ethics Committee of the University of Antwerp (B300201214328). Ethylenediaminetetraacetic acid (EDTA)-plasma from the healthy control group was centrifuged at 1200 x g for 15 minutes at 4 °C and stored at – 80 °C until analysis.

## Cytokine determination

Tumor necrosis factor (TNF) α, interleukin (IL)-1 receptor antagonist (IL-1RA), IL-1β, IL-6, IL-8 and IL-10 were analyzed batchwise in the EDTA plasma of the septic shock patients by a simultaneous Luminex assay according to the manufacturer’s instructions (Milliplex; Millipore, Billerica, MA).

## Statistical analysis

**Comparison of enzyme activities:** The comparison of the enzyme activities between the healthy controls and the non-septic shock ICU control group was carried out using a Mann-Whitney U test. Since a hypothesis test does not prove that the two control groups are equivalent, we additionally carried out a multivariate analysis (principal component analysis (PCA)) that lumps all information from the 4 original variables (PREP, PRCP, FAP and DPP4) into a new set of variables, referred to as principal components (PCs). The information is rearranged, constructing new continuous variables (PC1, PC2, PC3 and PC4), so that the information in the first PCs is maximized. One patient in the healthy control group had a very high PREP enzymatic activity and was removed from the analysis. During the measurement it was noticed that the sample was hemolyzed and it is known that PREP activity can increase due to lysis of platelets and red blood cells during sample preparation [1].

**Association analysis:** To model the influence of the enzyme activities (at day 1, 3, 5, 7) on the longitudinally measured parameters (measured on the same days), linear mixed models were fitted. Enzyme activity, time (categorical) and the interaction between them were entered as fixed effects. The significance of the interaction term (*p*Int) indicates whether the effect of the enzyme on the outcome is different between the 4 days. In case this interaction term was significant (*p*Int ≤ 0.01), simple linear regression models were fitted for the separate days. If *p*Int was not significant, indicating that the effect is the same over the four days, *p*Main was used, which means that there is an effect of the enzyme on the outcome when *p*Main ≤ 0.01. The parameters ventilation and dialysis were analyzed using generalized linear mixed models. To test the effect of the enzyme concentrations, recorded on day 1, on the SOFA score on day 1, we fitted simple linear regression models with the SOFA score on day 1 as dependent variable, and each of the enzyme concentrations as independent variable.

The effect of the enzymes on the outcomes measured once was modeled using either linear regression (continuous outcomes) or logistic regression (binary outcomes). The outcomes ‘length of intensive care stay’ and ‘duration of noradrenaline treatment’ were log transformed to obtain normality of the residuals. All statistical analyses have been performed using R (Version 3.1.2, R Core Development Team (2008)). Due to the multitude of statistical tests performed here, a *p*-value cutoff of 0.01 was used to declare significance.

# Results

## Enzyme activities healthy controls and non-septic shock ICU controls

The healthy controls had a mean age (SD) of 31 (13) years and consisted out of 17 women and 13 men. The enzymatic activities of both control groups can be found in S1 Fig. None of the enzymatic activities significantly differed between the two control groups (Mann-Whitney U test, *p* > 0.05). Principal component analysis (see S2 Fig) starting from the original variables PREP, PRCP, DPP4 and FAP showed that the first two PCs (PC1 and PC2), take up 76% of all the variance in the dataset. Plotting PC1 versus PC2, and differentially labeling the observations from ICU controls and healthy controls, did not show any clustering between the observations from these two datasets. There is no indication that the ICU observations would be any different from the healthy controls and this for all 4 enzymes. This is reassuring that the differences seen between the ICU control group and the septic shock patients, are indeed due to the disease state of the septic shock patients and not due to the underlying disease or surgery of the ICU control group.

In the ICU control group, the distribution of the PREP activities shows a much larger variance compared to the healthy controls.

## Relation between longitudinally measured parameters and enzyme activities

To model the effect of the 4 enzymes on several longitudinally measured parameters, we fitted linear mixed models, with each of the enzymes as explanatory variables and the parameter as outcome. The day of the recording was entered as covariate, and we also tested for the interaction between recording day and enzyme activity. This latter term tests whether the effect of the enzyme on the outcome parameter is uniform across the 4 recording days. For 2 of the longitudinally measured parameters, this interaction term was significant, indicating that the effect of the enzyme was day-dependent, while for others the effect was the same across the four time points. An overview of all studied parameters can be found in Table 1 in the main text. Day-dependent effects were found for PRCP on total bilirubin and TNFα (S3A Fig). Studying the enzyme effect on the separate days showed that PRCP was positively associated with total bilirubin on each measured day. On days 1, 5 and 7 the *p*-value was ≤ 0.05 (day 1 R² = 0.28, day 5 R² = 0.56, day 7 R² = 0.53). The association was even more pronounced on day 3 (*p* ≤ 0.001; R² = 0.76). The positive association between PRCP activity and TNFα was limited to days 1 and 3 (*p* ≤ 0.001; day 1 R² = 0.26, day 3 R² = 0.29). Several enzymes had an effect that was uniform across the days (S3B Fig (*p*Main) and S3C Fig (R²)). Some of these positive associations are quite significant, such as PRCP and PREP with the noradrenalin infusion rate to maintain an acceptable mean arterial blood pressure, PRCP with the serum lactate level, PREP with IL-1RA and PREP with intestinal fatty acid-binding protein (I-FABP).

Additionally, we fitted generalized linear mixed models to test if the enzymes were associated with the necessity of dialysis or ventilation, but no significant associations were found.

## Relation between outcomes measured once and enzyme activities

An overview of the studied parameters can be found in Table 1 in the main text. A significant association could only be found between DPP4 and the length of hospital stay (*p* = 0.002). Stepwise backward elimination resulted in a reduced regression model with the DPP4 activities of day 3 and day 5 as explanatory variables, explaining 27% of the variance (R²) in the length of the hospital stay.

# References

1. Bracke A, Van Elzen R, Van Der Veken P, Augustyns K, De Meester I, Lambeir A-M. The development and validation of a combined kinetic fluorometric activity assay for fibroblast activation protein alpha and prolyl oligopeptidase in plasma. Clin Chim Acta. 2019;495:154–60.

# List of abbreviations

AUC: area under the curve; APACHE II: Acute Physiology and Chronic Health Evaluation II; CI; confidence interval; DPP4: dipeptidyl peptidase 4; DTT: dithiothreitol; EDTA: ethylenediaminetetraacetic acid; FAP: fibroblast activation protein α; Gly-Pro-*p*NA: glycyl-prolyl-*para­*nitroanilide; ICU: intensive care unit; I-FABP: intestinal fatty-acid binding protein; IFNγ: interferon γ; IL: interleukin; IL-1RA: interleukin-1 receptor antagonist; IQR: interquartile range; LR-: negative likelihood ratio; LR+: positive likelihood ratio; MAP: mean arterial pressure; NA: not applicable; NIR, noradrenalin infusion rate; NPV: negative predictive value; PaO_2_/FiO_2_ ratio: ratio of arterial oxygen partial pressure to fractional inspired oxygen; PC: principal component; PCA: principal component analysis; PPV: positive predictive value; PRCP: prolylcarboxypeptidase; PREP: prolyl oligopeptidase; ROC: receiver operating characteristic; TNFα: tumor necrosis factor α; U/L: units per liter; Z-Gly-Pro-AMC: N-benzyloxycarbonyl-Gly-Pro-7-amido-4-methylcoumarine; Z-Pro-Phe: N-benzyloxycarbonyl-Pro-Phe.
